# Left Functional Pneumonectomy Caused by a Very Rare Giant Intrathoracic Cystic Lesion in a Patient with Gorham–Stout Syndrome: Case Report and Review of the Literature

**DOI:** 10.1155/2018/2406496

**Published:** 2018-04-12

**Authors:** Nikolaos Tasis, Ioannis Tsouknidas, Argyrios Ioannidis, Konstantinos Nassiopoulos, Dimitrios Filippou

**Affiliations:** ^1^Department of Anatomy and Surgical Anatomy, Medical School, National and Kapodistrian University of Athens, Athens, Greece; ^2^Hopital Daler, Fribourg, Switzerland; ^3^Department of Surgical Oncology, Laparoscopic Surgery and Laser Surgery, N Athinaio Hospital, Athens, Greece

## Abstract

Gorham–Stout syndrome is an uncommon entity, with few cases reported in bibliography. It consists of osteolytic manifestations affecting various bones and replacing them with lymphangiomatous tissue. With pathophysiology unknown, Gorham–Stout disease affects also cardiorespiratory system usually causing lytic lesions to the bones of the thoracic cage or directly invading the thoracic duct. This is a case report of a unique respiratory manifestation of the disease and a review of its cardiorespiratory complications.

## 1. Introduction

Gorham–Stout syndrome or vanishing bone disease is a rare entity with very few cases reported in global bibliography. Gorham et al. in 1954 [1] and Gorham and Stout in 1955 [2] firstly described this uncommon form of massive osteolysis. Till today, etiology and pathophysiology of the disease are unknown. The syndrome consists of replacement of normal bone by nonneoplastic vascular tissue, via overgrowth of lymphatic vessels, resulting in an “invisible” hypervascular fibrous bone [3]. The prognosis varies, with some patients achieving stabilization, while others, especially if complicated with pleural effusion, showing high mortality rates [4]. We present a case report of a unique thoracic presentation of Gorham–Stout syndrome and a detailed review of the literature concerning respiratory manifestations and complications of the disease.

## 2. Case Presentation

In 1997, female patient, 24 years old, presented intense lower back pain. Lumbar spine CT revealed multiple osteolytic lesions in lumbar vertebrae. Further investigation by attending physicians ruled out neoplastic infiltration. Clinical and laboratory exams were normal, except for slightly decreased calcium levels of unknown origin, with normal levels of PTH, so the patient was treated conservatively with NSAIDs and careful medical advice.

In May of 2004, the patient discovered a nodule in left thyroid lobe, which gradually increased in size. Biopsy revealed follicular thyroid cancer, and a total thyroidectomy was performed. Histological exam showed papillary and follicular thyroid cancer. Histological preparation did not consist of any parathyroid gland, although patient suffered from severe hypocalcaemia for many years postoperatively until utterly controlled by attending endocrinologist with administration of calcium and salmon calcitonin. In September of 2004, a large cystic mass was detected in anterior cervical surface, left of the clavicle. Ultrasound revealed a cyst with 4 cm diameter which was surgically removed. Histological exam suggested lymphatic cyst.

In 2007, a relapse was observed and was US monitored for the next months. In 2008, the patient was referred to our department for further investigation. Clinical examination showed the palpable mass in the lower anterior section of the cervix, with 3 cm diameter, increasing in size. Lab tests were normal apart from CPK (250 IU/L), serum phosphorus (5,6 mg/dl), and serum calcium (7,8 mg/dl). Thoracic and Cervical MRI reveal a substantial cystic mass (12,5 × 2,2 × 2,3 cm) in anterior mesothorax. Two similar masses were detected one paratracheal (2,6 × 4 cm) and one in the lower cervix (2,4 × 2 cm), all communicating. Lower and upper abdomen CT showed multiple cystic lesions of the spleen, while full MRI check revealed no change in cystic lesions of lumbar vertebrae, with several similar lesions in whole vertebrae column, the clavicle and both femurs. FNA procedure took place and the sample proved to be lymph. FNA in combination with CT and MRI results indicated Gorham–Stout disease.

Patient continued receiving conservative therapy with calcium and salmon calcitonin and after six months calcium levels became normal and bone density reached normal range, improving by far patient's quality of life. Mesothorax and spinal cystic lesions were reevaluated regularly and no change in size was noted.

The next two years patient underwent two incidents which were impossible to properly diagnose. The first consisted of an intense headache, which lasted for 15 days during patient's vacation, with following improvement and the second involved a vision disorder which was examined by an ophthalmologist with no pathology defined.

**Figure 1:**
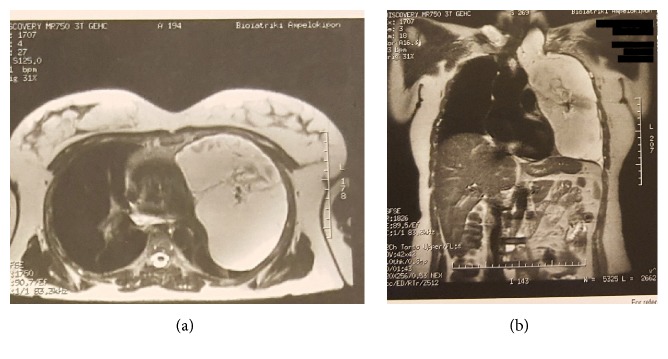
A giant cystic lesion occupying almost completely the left side of thorax causing functional pneumonectomy.

**Figure 2:**
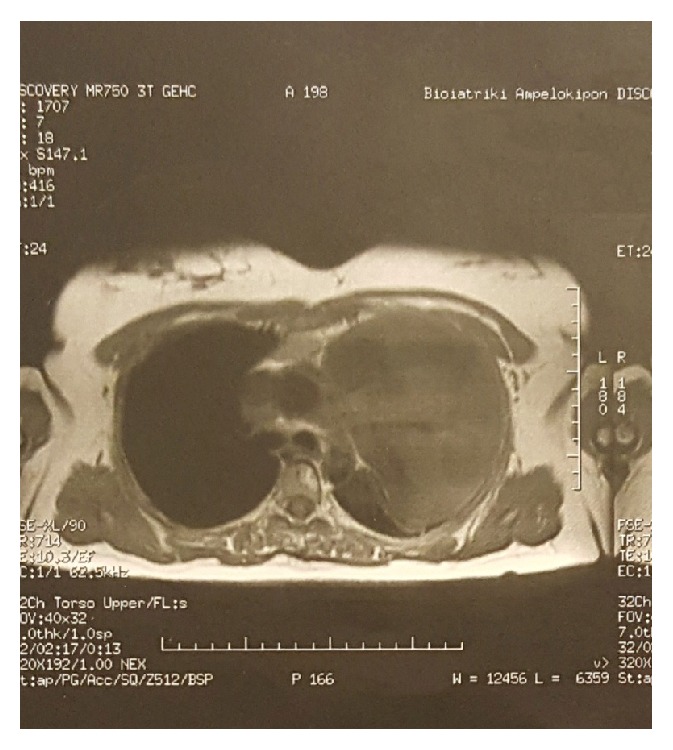
MRI of the intrathoracic cystic lesion. Osteolytic lesion in the thoracic vertebrae also obvious.

However, in 2010, the patient was admitted due to intense headache, gradually increasing, affecting patient's quality of life. Although examination revealed no abnormal findings, the patient was deteriorating. A lumbar puncture took place and showed low cerebrospinal fluid (CSF) pressure (<6). Intravenous fluids were administrated and, later, patient improved and was discharged, only to come two days later with severe temporal and cervical cephalalgia and vertigo. Otorhinolaryngological examination was not pathological and patient was treated conservatively with Sibellium, Lonarid-N, and Tramal tablets. Patient improved but further examination took place. Full-body 3D CT scan with intrathecal radioactive polymer infusion revealed the following: (1) incomplete herniation of the brain stem probably due to low CSF pressure; (2) dilation of endothoracic cyst, with more than 20 cm diameter, fully compressing left lung causing functional left pneumonectomy; and (3) possible communication of CSF with mesothoracic cystic mass (Figures 1(a), 1(b), and 2).

Surgical excision was undertaken. Patient underwent thoracoscopic excision of the gargantuan cystic mass, with ligation of major lymph vessels, conserving major thoracic duct. No connection between the cyst and spinal cavity was detected, possibly because during the excision procedure the connection was ligated or shut. Patient had a smooth postoperative course and recovery.

## 3. Discussion

Gorham–Stout syndrome (GSS), also known as Gorham's syndrome, idiopathic massive osteolysis, disappearing bone, disease or phantom bone disease, is a rare skeletal disorder, characterized by osteolysis of various bones accompanied by proliferative angiomatosis. Almost 180 years have passed since Jackson first presented, in 1838, the case of an 18-year-old boy with idiopathic osteolysis of his right humerus [62]. In 1955, Gorham and Stout, after concentrating and studying all previous similar cases, described “a syndrome of progressive osteolysis associated with an angiomatosis proliferation of blood or lymphatic vessels” [2]. Although over 200 cases have been reported to date [63], there is still a lot to learn about this syndrome.

Gorham–Stout syndrome provokes osteolytic damage to one or multiple bones. It can affect any age, but most commonly children and young adults. Special correlation with specific race, sex, geography, or hereditary pattern has not been identified yet [64]. The shoulder and the pelvis are the most common sites to be affected according to Patel [3]. Femur as more possible first presentation is described by Ruggieri et al. [65] and Hu et al. [66]. Cases of phantom bone disease to the skull, mandible, maxillofacial skeleton, spine, scapula, clavicle, ribs, sternum, humerus, hand, and foot have also been described [3]. In our case lumbar osteolytic lesions may be considered the first sign of Gorham–Stout syndrome. Our patient experienced intense low back pain at the age of 22. The symptom was treated as common back pain and although lesions of lumbar spine were detected, no further investigation was required since neoplastic etiology was ruled out and laboratory tests were normal. Pain was relieved with NSAIDs and the patient continued everyday activity without symptoms for years. Pain is considered to be a frequent atypical symptom of GSS [64], mainly because of lymphangiomatous infiltration of bones. Maillot et al. [67] state that vertebral primary involvement is rare (10%) and associated with a poor prognosis. However, patients tend to be undiagnosed until pathological fracture takes place even if localized pain is present [65]. The fact that Gorham–Stout progression may be asymptomatic reinforces our belief of lumbar spine involvement in our case.

In the course of the case, two years after thyroidectomy, a cystic mass was spotted in lower anterior cervix of the patient. It was identified as a lymphangiomatous cyst and was surgically excised. Six months later, a relapse caused the referral of the patient to our department only to diagnose one large mesothoracic cyst (12,5 × 2,2 × 2,3 cm) with two smaller and one paratracheal (2,6 × 4 cm) cysts and one in the lower cervix (2,4 × 2 cm). In addition, osteolytic lymphangiomatous lesions, common Gorham–Stout syndrome's manifestations [64], were located in whole vertebrae column, the clavicle, and both femurs as well as in spleen. Patient was treated conservatively with calcitonin and regularly checked. Cysts were monitored and there was no change in size until patient had a thorough investigation for intense cephalalgia. Only then did we come across a unique finding. CT revealed dilation of endothoracic cyst, more than 20 cm in diameter, fully compressing left lung causing functional left pneumonectomy. Also, there was incomplete herniation of the brain stem due to low CSF pressure, probably because of possible communication of CSF with mesothoracic cystic mass, which could not be verified thoracoscopically.

Disease of the bones of the thoracic cage often presents with pleural effusion and/or chylothorax. Chylothorax is present up to 17% of the patients [50] and increases the rate of mortality and morbidity, especially if patients do not undergo surgical intervention [68]. There are 67 confirmed cases of Gorham–Stout syndrome with cardiorespiratory invasion found in the literature (Table 1). Chylothorax was present in 60 of them (89,55%) and was the major presenting symptom in the thorax. In literature we found, also, two cases of nondescript pleural effusion [4, 56], one case of hemothorax [29], one case with hematoma [29], and three cases with bloody pleural effusion [32, 34, 47]. One unusual case of duropleural fistula [47] was noted as well. Concerning more rare cardiac presentations, there were two cases with chylotamponade [41, 52], one with hemochylopericardium [46], and two cases of nondescript pericardial effusion [4, 44] reported in literature. In these 67 cases we only encountered 2 cases of lymphangiomatous cysts [37, 61], one in the mediastinum and one located in right anterior chest wall. Our case is the third with cystic presentation of Gorham–Stout syndrome. However, the fact that the massive lymphangiomatous cyst provoked a functional left pneumonectomy due to its size and position in the mesothorax has not been described previously in Gorham–Stout disease. Usually, the cysts are found and dealt with at early stages or erupt into the pleural cavity causing chylothorax. In this case the cyst reached 20 cm in diameter, caused left functional pneumonectomy, and was identified randomly after CT scan. Our patient underwent surgery and had an uncomplicated recovery. Mortality in Gorham–Stout disease with thoracic cage presentation as reported in the literature is high. Out of 67 cases, 22 did not survive (32,8%). Surgical ligation, pleurodesis, and other surgical or radiological treatments seem to improve survival for patients with these complications [68].

## 4. Conclusion

Gorham–Stout syndrome is an uncommon condition. With etiology unknown, diagnosis and treatment remain a challenge. Osteolytic lesions and/or pathological fractures should raise clinical suspicion for GSS. Attention is needed, especially when respiratory complications are present. This case offers a new presentation of the disease and along with past and future cases may contribute to a deeper understanding of this extraordinary clinical entity, the Gorham–Stout Syndrome.

## Figures and Tables

**Table 1:** Presentation of all cases of patients with respiratory manifestations of Gorham–Stout syndrome reported in the literature.

Number	Author	Year	Age/sex	Hemithorax involved	Pathology	Outcome
(1)	Gorham et al. [1]	1954	16/*Μ*	R	Chylothorax	Died

(2)	De seze and Hubault [5]	1956	29/F	Bilateral	Chylothorax	N/A

(3)	Jones et al. [6]	1958	28/M	Bilateral	Chylothorax	Died

(4)	Koblenzer and Bukowski [7]	1961	4/F	R	Chylothorax	Died

(5)	Tucker [8]	1967	11/M	Bilateral	Chylothorax	Survived

(6)	Kolpakova and Iaroshevskaia [9]	1967	12/F	Bilateral	Chylothorax	Died

(7)	Vanetti et al. [10]	1968	5/F	L	Chylothorax	Survived

(8)	Morphis et al. [11]	1970	9 mo/M	Bilateral	Chylothorax	Died

(9)	Touraine et al. [12]	1970	49/M	L	Chylothorax	Survived

(10)	Takamoto et al. [13]	1971	21/M	R	Chylothorax	Survived

(11)	Gutierrez and Spjut [14]	1972	4/M	Bilateral	Chylothorax	Died

(12)	Berberich et al. [15]	1975	3.5/M	Bilateral	Chylothorax	Survived

(13)	Noziska et al. [16]	1974	14/M	Bilateral	Chylothorax	Died

(14)	Fessard et al. [17]	1974	2/F	R	Chylothorax	Died
(15)			3/F	Bilateral	Chylothorax	Died

(16)	Patrick [18]	1976	30/M	L	Chylothorax	Survived

(17)	Rousselin et al. [19]	1977	30/F	L	Chylothorax	Died

(18)	Feigl et al. [20]	1981	26/F	Bilateral	Chylothorax	Survived

(19)	Young et al. [21]	1983	2/M	R	Chylothorax	Survived

(20)	Pedicelli et al. [22]	1984	18/F	L	Chylothorax	Survived

(21)	Brown et al. [23]	1986	30/M	Bilateral	Chylothorax	Died

(22)	Choma et al. [24]	1987	23/M	R	Chylothorax	Survived

(23)	Hejgaard and Olsen [25]	1987	9/M	R	Chylothorax	Survived

(24)	Joseph and Bartal [26]	1987	7/F	R	Chylothorax	Died

(25)	Marymont [27]	1987	7/F	R	Chylothorax	Died

(26)	Romero et al. [28]	1989	61/M	R	Chylothorax	Survived

(27)	Meller et al. [29]	1993	7 mo/M	R	Hemothorax	Survived
(28)			14/M	R	Hematoma	Survived

(29)	Tie et al. [30]	1994	18/M	L	Chylothorax	Survived

(30)	Drewry et al. [31]	1994	13/M	L	Chylothorax	Survived

(31)	Ng and Wang [32]	1995	63/F	R	Pleural effusion (Blood stained serous fluid)	Died

(32)	Riantawan et al. [33]	1996	27/M	Bilateral	Chylothorax	Died

(33)	McNeil et al. [34]	1996	21/M	R	Pleural effusion (Bloodstained fluid)	Survived

(34)	Aoki et al. [35]	1996	19/F	R	Chylothorax	Survived

(35)	Chavanis et al. [36]	2001	45/F	L	Chylothorax	Survived

(36)	Yoo et al. [37]	2002	38/M	Bilateral	Chylothorax, Mediastinal cystic mass (with turbid pinkish fluid)	Survived

(37)	Fujiu et al. [38]	2002	15/M	Bilateral	Chylothorax	Died

(38)	Miller [39]	2002	2/M	R	Chylothorax	Died

(39)	Lee et al. [40]	2002	25/F	R	Chylothorax	Survived

(40)	Swelstad et al. [41]	2003	31/F	R	Chylothorax, chylotamponade	Survived

(41)	Lee et al. [42]	2003	6/F	N/A	Chylothorax	Died
(42)			9/F	N/A	Chylothorax	Died

(43)	Fontanesi [43]	2003	M	R	Chylothorax	Survived

(44)	Takahashi et al. [44]	2005	2/F	L	Chylothorax, Pericardial effusion	Survived

(45)	Kren et al. [45]	2005	7/M	L	Chylothorax	Survived

(46)	Duffy et al. [46]	2005	31/F	Bilateral	Chylothorax, hemochylopericardium	Survived

(47)	Agrawal et al. [47]	2006	25/F	L	Bloody pleural effusion, DPF	Survived

(48)	Pfleger et al. [48]	2006	18/M	R	Chylothorax	Survived

(49)	Yildiz et al. [49]	2009	6/M	Bilateral	Chylothorax	Survived

(50)	Kose et al. [50]	2009	9/F	R	Chylothorax	Survived

(51)	De Smet et al. [51]	2010	8/M	L	Chylothorax	Survived

(52)	Wijesinghe et al. [52]	2010	50/F	L	Chylothorax, Chylotamponade	Survived

(53)	Kuriyama et al. [53]	2010	16/F	L	Chylothorax	Survived

(54)	Deveci et al. [54]	2011	6/M	Bilateral	Chylothorax	Died

(55)	Brodszki et al. [55]	2011	2.5/M	R	Chylothorax	Survived
(56)			4/F	Bilateral	Chylothorax	Survived

(57)	Min-Wen et al. [56]	2012	5/F	L	Pleural effusion	Survived

(58)	Hopman et al. [57]	2012	12 mo/M	R	Chylothorax	Died

(59)	Noda et al. [58]	2013	15/M	R	Chylothorax	Survived

(60)	Jayaprakash et al. [59]	2013	9/F	Bilateral	Chylothorax	Died

(61)	Maillot et al. [60]	2014	30/F	L	Chylothorax	Survived

(62)	Liu et al. [4]	2014	32/F	Bilateral	Pleural effusion, Pericardial effusion	Survived
(63)			15/M	Bilateral	Chylothorax	Survived
(64)			32/M	Bilateral	Chylothorax	Survived
(65)			22/F	L	Chylothorax	Survived
(66)			23/M	R	Chylothorax	Survived

(67)	Davalos et al. [61]	2015	9/F	R	Large cystic mass, chylothorax	Survived
